# Fabrication and Characterization of Poly (vinyl alcohol) and Chitosan Oligosaccharide-Based Blend Films

**DOI:** 10.3390/gels7020055

**Published:** 2021-05-06

**Authors:** Dilshad Qureshi, Ayasharani Sahoo, Biswaranjan Mohanty, Arfat Anis, Viktoryia Kulikouskaya, Kseniya Hileuskaya, Vladimir Agabekov, Preetam Sarkar, Sirsendu Sekhar Ray, Samarendra Maji, Kunal Pal

**Affiliations:** 1 Department of Biotechnology and Medical Engineering, National Institute of Technology Rourkela, Rourkela 769008, India; dilshadq786@gmail.com (D.Q.); ayasha11sahoo@gmail.com (A.S.); sirsendu.iitkgp@gmail.com (S.S.R.); 2 Institute of Pharmacy and Technology, Salipur 754202, India; biswaranjanm5@gmail.com; 3 SABIC Polymer Research Center, Department of Chemical Engineering, King Saud University, Riyadh 11451, Saudi Arabia; aarfat@ksu.edu.sa; 4 The Institute of Chemistry of New Materials, National Academy of Sciences of Belarus, 220141 Minsk, Belarus; kulikouskaya@gmail.com (V.K.); k_hilevskay@mail.ru (K.H.); mixa@ichnm.by (V.A.); 5 Department of Food Process Engineering, National Institute of Technology, Rourkela 769008, India; preetamdt@gmail.com; 6 Department of Chemistry, SRM Institute of Science and Technology, Kattankulathur, Chennai 603203, India

**Keywords:** poly (vinyl alcohol), chitosan oligosaccharide, blends, films, semicrystalline, drug delivery

## Abstract

In the present study, we report the development of poly (vinyl alcohol) (PVA) and chitosan oligosaccharide (COS)-based novel blend films. The concentration of COS was varied between 2.5–10.0 wt% within the films. The inclusion of COS added a brown hue to the films. FTIR spectroscopy revealed that the extent of intermolecular hydrogen bonding was most prominent in the film that contained 5.0 wt% of COS. The diffractograms showed that COS altered the degree of crystallinity of the films in a composition-dependent manner. As evident from the thermal analysis, COS content profoundly impacted the evaporation of water molecules from the composite films. Stress relaxation studies demonstrated that the blend films exhibited more mechanical stability as compared to the control film. The impedance profiles indicated the capacitive-dominant behavior of the prepared films. Ciprofloxacin HCl-loaded films showed excellent antimicrobial activity against *Escherichia coli* and *Bacillus cereus*. The prepared films were observed to be biocompatible. Hence, the prepared PVA/COS-based blend films may be explored for drug delivery applications.

## 1. Introduction

Poly (vinyl alcohol) (PVA) is a thermoplastic synthetic polymer that is prepared either by partial or complete hydrolysis of polyvinyl acetate [1,2,3]. The polymer is readily solubilized in water and its solubility characteristics are dependent on molecular weight, particle size distribution, and crystallinity of the polymer chains [1,2]. Certain factors such as molecular weight, concentration, and hydrolysis degree of PVA have a major effect on the performance of PVA-based polymeric architectures [4]. The polymer is benign to living tissues, harmless, non-carcinogenic, non-toxic, biodegradable, and biocompatible [3]. PVA has been approved by the Food and Drug Administration (FDA) to be utilized in pharmaceutical industries [5]. On the other hand, owing to its certain features like bio-inertness and compatibility, PVA has found implications in different medical fields, e.g., drug delivery, hemodialysis, nanofiltration, and implantable medical devices [6,7]. PVA is a hydrophilic polymer and therefore, exhibits excellent water retention properties apart from its good mechanical properties [8]. However, the major shortcoming associated with PVA-based materials is their swelling and fast dissolution when the polymeric architectures of PVA come in contact with water [9]. As per the literature, the mechanical and thermal attributes of the PVA-based polymeric architectures can be improved by crosslinking the polymer using irradiation or bi/multi-functional reactive group-containing chemical agents (e.g., glutaraldehyde (GTA)) [9,10]. Over the years, various GTA crosslinked PVA-based architectures (such as hydrogels, films, nanofibers, and nanoparticles) have been explored for drug delivery, tissue engineering, wound healing, and dosimetry applications [11,12,13,14,15,16,17,18,19,20]. Furthermore, owing to the tissue-mimicking properties, PVA-based matrices have acquired peculiar attention as soft tissue phantoms in biomedical research for the substitution of real tissues in vitro [21,22,23]. PVA-based hydrogels have been utilized to fabricate soft contact lenses [24], artificial cornea [25], artificial pancreas [26], orthopedic implantations (intervertebral disc, artificial articular cartilage) [27], and artificial meniscus [28]. Recently, polymeric film matrices have demonstrated their potential as efficient drug dosage platforms due to their ability to: (a) enhance drug efficacy and the onset of drug action, (b) reduce dosage frequency, (c) improve drug retention, and (d) increase patient compliance [29,30]. Since PVA exhibits excellent film-forming capacity [31], many researchers have employed this property to develop PVA-based films for ophthalmic [32], oral [33], buccal [34], topical [5], transdermal [35], and vaginal [36] drug delivery applications. Furthermore, PVA has also been known to synthesize blends with natural polysaccharides (e.g., chitosan, alginate, starch, and tara gum) to prepare films with improved mechanical and thermal properties [37,38]. In a recent study conducted by Engelke and coworkers (2018), polymeric films of pristine PVA, PVA/carboxymethyl cellulose, and PVA/carbomer blends were prepared, characterized, and were used as the needle-free delivery method for facilitating the delivery of macromolecules and nanoparticles across the laser microporated skin [39]. 

Chitosan is a linear polysaccharide that is chemically converted from chitin (natural polymer obtained from shells of shrimps and other sea crustaceans) [40,41,42]. This polymer has been exploited for ages in the biomedical arena to fabricate various products like hemostatic bandages and drug delivery systems [42,43,44]. However, chitosan has several limitations, such as low water solubility and slow water absorption capacity [45,46]. Hence, to overcome the aforesaid limitations, various water-soluble chitosan derivatives (e.g., chitosan oligosaccharide, chitosan lactate, chitosan succinate, chitosan glutamate, etc.) have been proposed. Chitosan oligosaccharide (COS) is a low molecular weight product of chitosan, obtained by its enzymatic or chemical degradation [47]. Due to its excellent water solubility properties [48,49] and high biological activity, COS is easy to handle as compared to chitosan [50]. COS is widely used in the biomedical, food, and pharmaceutical industries [51]. The significant biological properties of COS include antitumor, antibacterial (against *Escherichia coli*, *Bacillus cereus*, and *Staphylococcus aureus*), antifungal, anti-inflammatory [45,50], antioxidant, and non-toxic properties [47]. Additionally, COS can enhance immunity by activating a variety of immune responses. The antimicrobial activity of COS can protect the wounds against infections, improve moisture penetrability, and promote cell proliferation. These properties of COS are responsible for accelerating the healing of wounds [52]. Moreover, numerous COS-based systems have been developed over the years as ocular [53], transdermal [54], anti-cancer [46], and buccal [49] drug delivery carriers. For instance, recently Kumar and coworkers (2019) fabricated COS-based blended films as buccal drug delivery systems. The authors reported that COS could be well-blended with PVA and alginate. COS-PVA films exhibited superior mucoadhesive properties and elasticity as compared to COS-alginate films [49]. Unfortunately, the effect of a variable proportion of COS and PVA on the physicochemical aspects of the composite films was not assessed by the research group. This limitation was addressed by Mahato et al. (2020) whose research group prepared PVA/COS-based hydrogels for the purpose of drug delivery. The COS concentration within the hydrogels was maintained within the range of 0.25–2% *w*/*v*. In vitro release behavior of Lomefloxacin (model drug) suggested the potency of the PVA/COS films as drug delivery matrices [47]. As far as the authors are aware, only Kumar et al. (2019) [49] and Mahato et al. (2020) [47] have studied PVA/COS hydrogel-based drug delivery systems. However, the scope for analyzing the impact of COS content on the physicochemical and biological attributes of the PVA matrices was observed to be severely limited in the aforementioned research works. 

Taking a note from the above discussion, herein, we aimed to prepare chemically crosslinked PVA/COS-based films by employing the conventional solvent casting method. In our study, the concentration of COS gel in PVA was varied between 2.5–10% *w*/*w*, which was considerably higher as compared to its concentration in previously reported COS-containing hydrogel systems [47,49]. Various physicochemical analyses such as infrared spectroscopy, X-ray diffraction, electrical impedance, thermal, and mechanical analysis were performed to assess the variations in the properties of the prepared films as a function of COS concentration. Moreover, the biological attributes of the films such as antimicrobial activity and hemocompatibility were also investigated. The prepared PVA/COS films were used as carriers for the model antibiotic ciprofloxacin hydrochloride (CPH). It is expected that the novel PVA/COS films could be explored as custom-tailorable drug carriers for controlled drug delivery applications. 

## 2. Results and Discussion

### 2.1. Preparation of the Films

**Figure 1:**
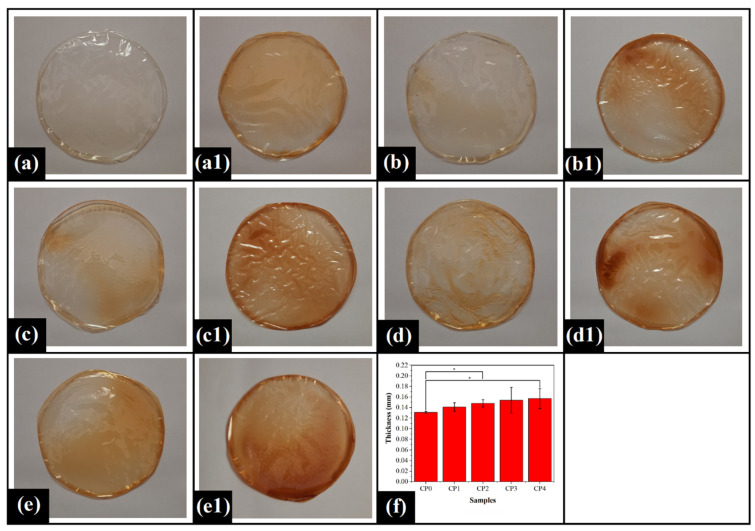
Pictographs of the prepared films: (**a**) CP0, (**a1**) CP0D; (**b**) CP1, (**b1**) CP1D; (**c**) CP2, (**c1**) CP2D; (**d**) CP3, (**d1**) CP3D; (**e**) CP4, (**e1**) CP4D; and (**f**) Thickness of the pristine films (mean ± standard deviation). The symbol * denotes the significant differences at *p* < 0.05 level.

The composite films were prepared by the solution casting method. For this purpose, a homogeneous mixture of PVA solution and COS gel was used. The mixing process mediated the complete solubilization of COS gel within the PVA solution, followed by the dilution of the mixture with water. The resulting diluted mixture was a light, yellowish-colored solution. The obtained mixture was degassed, and subsequently, the crosslinking agent was added. Thereafter, the mixtures were transferred into Petri plates for drying. Post-drying, the polymeric films were taken out of the Petri plates. The control film (CP0) did not contain any COS gel and hence, appeared transparent and colorless (Figure 1). PVA/COS films (CP1-CP4) were translucent and showed a brownish color (Figure 1). The average thickness of the films varied in the range of 0.13–0.18 mm. The thickness of CP0 was significantly lower than CP2 and CP4 (*p* < 0.05). However, the variation in the thickness of CP0, CP1, and CP3 was insignificant (*p* > 0.05). The average thickness of the films was increased with enhancing COS content. The increment in the thickness of PVA/COS films might be attributed to the water-holding capacity of the chitosan molecules [55]. The higher standard deviation values in PVA/COS films indicated significant variations in the thickness of these films. The transparency of the films was reduced with the rise in COS concentration. Moreover, the PVA/COS films became darker, with an increment in COS content [56] (Figure 1). It is noteworthy that the drug-loaded films possessed a much darker brownish color as compared to the films which were not loaded with the drug. All the films were flexible and had smooth surfaces without any cracks and pores.

### 2.2. Fourier Transform Infrared (FTIR) Spectroscopy Analysis 

**Figure 2:**
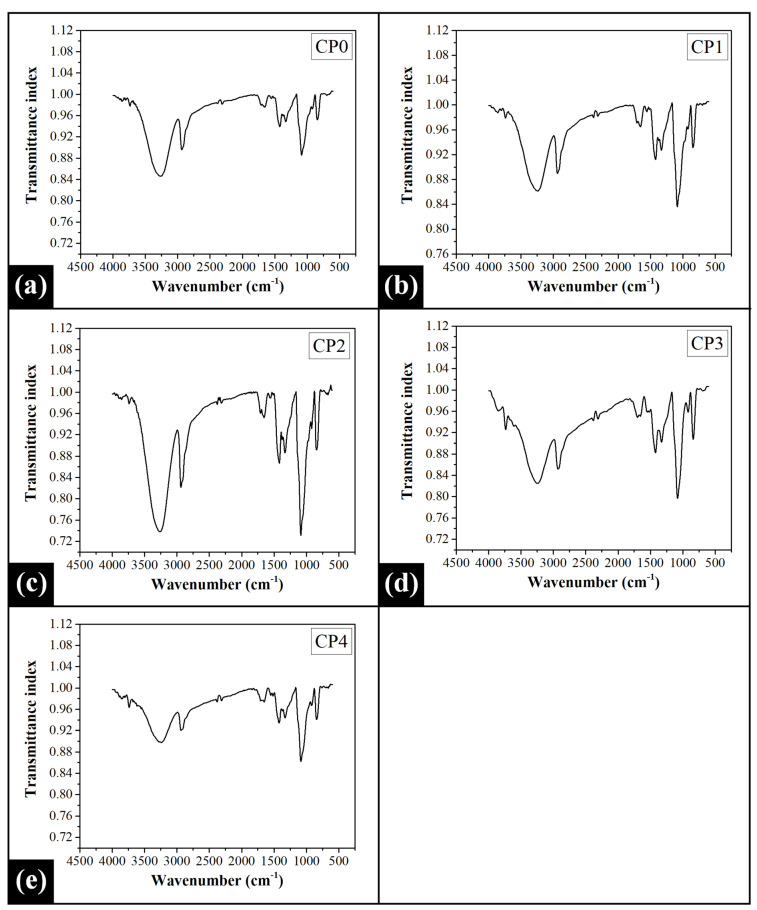
ATR-FTIR spectra of the prepared films: (**a**) CP0, (**b**) CP1, (**c**) CP2, (**d**) CP3, and (**e**) CP4.

The control film (CP0) showed a small absorption band at 3734 cm^−1^ in its FTIR spectrum, which is due to the presence of free or non-bonded hydroxyl groups [57] (Figure 2). The most prominent peak observed in the spectrum of CP0 was a broad O-H stretching band that was observed at 3269 cm^−1^. This suggested the occurrence of intra- and intermolecular hydrogen bonding among the -OH groups of the PVA chains [57]. A sharp peak located at 2936 cm^−1^ can be assigned to the asymmetric C-H stretching of the methylene (-CH_2_) groups [58]. A characteristic vibrational signal was obtained at 1708 cm^−1^ for the stretching of carbonyl (-C=O) functional groups. The presence of carbonyl groups can be attributed to the residual acetate moieties that remain after PVA production from the hydrolysis of polyvinyl acetate [59]. It has been reported that C=O stretching bands obtained in the range of 1700–1720 cm^−1^ are assigned to the hydrogen-bonded C=O groups [60]. The peak recorded at around 1652 cm^−1^ can be accredited to ν_δ_(H-O-H) bending vibration of water, which must have remained in the PVA control film after drying [61]. The vibrational signal corresponding to the bending of the -OH groups was recorded at 1422 cm^−1^ [62]. The sharp and strong peak that appeared at 1330 cm^−1^ could be ascribed to the CH-OH stretching vibration [63], while the signal corresponding to C-O stretching was noted at 1085 cm^−1^ [59]. A moderate band recorded at 842 cm^−1^ can be accredited to the C-C stretching vibration [64].

The addition of COS polysaccharide introduced significant variations in the FTIR spectra of the blended films (Figure 2). It can be observed that the sharp band assigned for free -OH groups blue-shifted to 3740 cm^−1^ by 6 cm^−1^ in CP1 and CP2. This signal shifted to 3736 cm^−1^ and 3738 cm^−1^ in CP3 and CP4, respectively. However, the apparent positional fluctuation of the peak maximum of the said vibrational signal was not significant. On an overall basis, the blue-shifting of the peak might have occurred due to the increment in hydroxyl groups with the addition of COS. The spectra of the PVA/COS blended films also demonstrated a broad peak that can be assigned to the O-H and N-H stretching vibrations [62]. As compared to CP0, this band red-shifted by 29 cm^−1^ in CP1 (3240 cm^−1^), while there was no significant shift in the peak position in the case of CP2 (3267 cm^−1^). As the concentration of COS was further increased, significant hypsochromic shift by 25 cm^−1^ and 29 cm^−1^ was observed in CP3 (3244 cm^−1^) and CP4 (3240 cm^−1^), respectively. This shift indicated the formation of new hydrogen bonds either within the PVA chains or between PVA and COS. It can also be speculated from the peak shift that the PVA/COS blends exhibited relatively more strong hydrogen bonding as compared to the control [65]. The sharp peak assigned for the C-H stretching of the alkyl groups was obtained at the same position of 2936 cm^−1^ in CP1 (2936 cm^−1^), CP2 (2938 cm^−1^), and CP4 (2936 cm^−1^) as that of CP0. However, this C-H stretching signal red-shifted by 10 cm^−1^ in CP3 (2926 cm^−1^). The vibrational signal assigned to C=O stretching demonstrated no significant shifting in its position (1708 cm^−1^) in CP1, CP2, and CP4. Interestingly, in CP3, the peak downshifted by 9 cm^−1^ to 1699 cm^−1^. The absorption band accredited to the amide-I (C=O vibration mode) was observed in the range of 1650–1655 cm^−1^ in PVA/COS films [66]. The band was overlapped with the ν_δ_(H-O-H) bending vibration signal (1652 cm^−1^) of the PVA, as observed in CP0. The peak assigned to the amide-I moiety was observed at an approximately similar location in CP1 (1652 cm^−1^), CP2 (1655 cm^−1^), and CP3 (1655 cm^−1^). A further increment in the COS concentration displaced this vibrational signal to a much lower wavenumber position (red-shifting) in CP4 (1650 cm^−1^). Hence, the interaction of PVA and COS through amide linkage formation was evident through this peak shifting. The characteristic band for N-H bending vibration (amide II) was also observed in all PVA/COS films [66]. It was located at 1555 cm^−1^ in CP1 while the signal blue-shifted in CP2 (1561 cm^−1^). The N-H bending vibration signal was located at the lowest wavenumber of 1548 cm^−1^ in CP3 while the same shifted to 1550 cm^−1^ in CP4. Apart from the above-mentioned peaks, the PVA/COS films also showed characteristic vibrational bands for -OH group bending (~1422 cm^−1^), CH-OH stretching (~1330 cm^−1^), C-O stretching (~1085 cm^−1^), and C-C stretching (~842 cm^−1^). No significant fluctuation in the position of these peaks was observed in the spectra of the PVA/COS films. 

In order to assess the extent of intra- and intermolecular hydrogen bonding between the components of the films, the area under the O-H stretching peak was calculated using Origin Pro software (Figure S1). The results showed that the control film exhibited a moderate area under the peak (AUP). The inclusion of COS in the PVA matrix abruptly decreased the AUP value in CP1. As the content of COS further increased in CP2, the AUP reached its highest value, which suggested a high degree of hydrogen bonding. A subsequent rise in COS content caused a corresponding decrease in the hydrogen bonding in CP3 and CP4. 

### 2.3. X-ray Diffraction (XRD) Analysis

**Figure 3:**
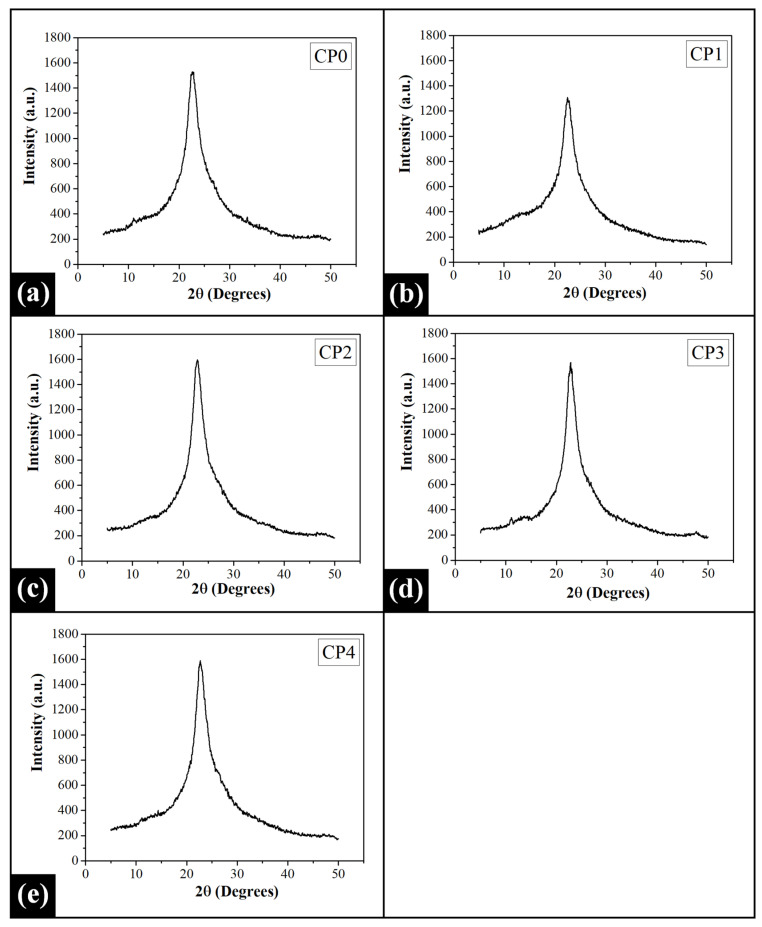
X-ray diffractograms of the prepared films: (**a**) CP0, (**b**) CP1, (**c**) CP2, (**d**) CP3, and (**e**) CP4.

The X-ray diffraction profile of the CP0 film demonstrated a sharp peak at 22.60° 2θ, which has been a representation of the crystalline nature of the PVA matrix (Figure 3) [67]. The influence of COS inclusion on the crystallinity of the PVA matrix can be observed from the diffraction profiles of PVA/COS films. The peak at 22.60° 2θ of the CP0 film displaced to a lower 2θ angle in CP1 (22.56° 2θ) while a shift to higher angles in CP2 (22.86° 2θ), CP3 (22.82° 2θ), and CP4 (22.70° 2θ). This shift strongly suggested the increment of the amorphous nature of the PVA matrix in CP1 while other PVA/COS films exhibited an increase in their crystalline nature. Moreover, the shift in peak location indicated the existence of some strong specific interactions among the functional groups of the PVA and COS. It was observed that the intensity of this major peak was higher in the PVA/COS films as compared to the control (CP0), except CP1. Among all the films, the intensity of the peak was observed to be the lowest in CP1. Hence, the domination of the crystalline phase in the PVA matrix was evident with an increment in COS concentration. The decrement in the peak intensity suggested that the polymer matrix in CP1 exhibited a lower crystalline phase. These results can be appropriately explained by taking into account the semicrystalline nature of the PVA matrix, where amorphous and crystalline regions co-exist in variable extents. The crystalline portions of the matrix are the compactly arranged polymer chains (crystallites), which are interspersed with the amorphously structured domains [68]. The percentage crystallinity of the PVA/COS films as compared to CP0 was estimated using the intensity values of the crystalline peak (Equation (1); Table S1). It was observed that the crystalline nature of the PVA matrix was –14.50% in CP1. The –ve sign suggests that there was a decrement in the crystallinity of CP1 as compared to CP0. The % increase in the crystallinity of CP2, CP3, and CP4 was 4.38%, 2.60%, and 3.91%, respectively. 

The major diffraction signal was deconvoluted using the Gauss function of Origin Pro software to assess the extent of the crystalline and amorphous nature of the films. The peak fitting results demonstrated the occurrence of four peaks (Figure S2). However, it can be observed that the main peak was constituted of an amorphous peak (green; peak A) and a crystalline peak (blue; peak B). Hence, the peak positions and full-width at half maxima (FWHM) values of these two resolved peaks were used for calculating the parameters such as d-spacing (Bragg’s law; Equation (2)), the crystallite size (D) (Scherrer’s formula; Equation (3)), and lattice strain (Equation (4)) (Table 1) [69]. The variations in the FWHM values of the peaks can be associated with the changes in the crystalline or amorphous nature of the films. The average FWHM value of the peak in CP0 was 4.491° 2θ. The incorporation of COS in the PVA matrix reduced the average FWHM in CP1 (4.452° 2θ), while the same increased to 4.606° 2θ in CP2. A further increment in COS content reduced the average FWHM to the lowest value (4.335° 2θ) in CP3, which can be attributed to the abundance of the high crystallinity of the composite matrix. Interestingly, in CP4, the average FHWM value of the peak again increased to 4.504° 2θ, which lied in the range of the average FHWM values of CP1 and CP2. 

Moreover, the height ratio of peak B and peak A $\left(\frac{B\;\left(\mathrm{low}\;\mathrm{FWHM}\right)}{A\;\left(\mathrm{high}\;\mathrm{FWHM}\right)}\right)$ was calculated to get an insight regarding the relative crystallinity of the films (Table S1). The relative crystallinity of the films increased in the order of CP0 (1.196) < CP1 (1.352) < CP4 (1.417) < CP2 (1.467) < CP3 (1.619). The calculated interplanar spacings also showed alterations with the addition of COS. The average d-spacing value of CP1 (4.550 Å) was comparatively larger as compared to that of CP0 (4.545 Å). A marked decrease in the d-spacing was observed in CP2 (4.512 Å). Interestingly, the interplanar spacing of CP3 (4.513 Å) was nearly equal to that of CP2, while a sudden rise in the average d-spacing was noticed in CP4 (4.528 Å). Furthermore, the average crystallite size (D) was also calculated. The average D-value of CP0 (3.310 nm) was moderate. The average size of the crystallites reduced in CP1 (3.295 nm) and CP2 (3.255 nm) with a corresponding increment in COS content. It can be observed that the crystalline regions of CP3 exhibited the largest crystallites (3.410 nm), which indicated that the crystallinity of the composite matrix in this blend film was high. In the case of CP4, a sudden decrement in average D-value (3.270 nm) was noticed. The introduction of lattice imperfections in the PVA crystals with the inclusion of COS was assessed by calculating the lattice strain parameter. The average lattice strain values of the films showed no substantial changes with the inclusion of COS in the PVA matrix. 

The intensity values of the resolved peak A and peak B were used to evaluate the degree of crystallinity (*X_c_*) of the films (Equation (5); Table S1) [70]. The intensity of the crystalline phase was observed to be higher in PVA/COS composite films (except CP1) as compared to the intensity values of the pristine film. The *X_c_* value of CP0 was 53.232%. In the case of blend films, the *X_c_* value gradually monotonously increased in CP1 (55.525%), CP2 (57.021%), and CP3 (58.773%). The *X_c_* showed a sudden decrement in CP4 (56.416%), which was quite expected from the average FWHM values. Hence, it can be speculated that the interaction of PVA with COS changed the amount of crystalline phase in the polymer matrix.

(1) $$\%\;∆\mathrm{crystallinity}=\frac{I_{\mathrm{cs}}-I_{\mathrm{cc}}}{I_{\mathrm{cc}}}\times 100$$

where % Δcrystallinity is the percentage change in crystallinity, I_cs_ is the intensity of the crystalline peak of a particular sample, and I_cc_ is the intensity of the intense peak in the control sample.

(2) nλ=2d.sinθ

where *λ* is the X-ray wavelength (1.79 Å), *θ* is the diffraction angle, *n* is an integer, and *d* is the d-spacing.

(3) $$D=\frac{k\lambda }{\beta \;cos\theta }$$

where *D* is the apparent size of the crystal, *θ* is the diffraction angle, *K* is a constant close to unity (i.e., 0.94), *β* is the FWHM (in radian), and *λ* is the wavelength (i.e., 1.79 Å).

(4) $$\varepsilon =\frac{\beta }{4\tan\theta }$$

where *ε* represents the strain of the material, *β* is the FWHM (in radian), and *θ* represents diffraction angle.

(5) $$X_{c}=\frac{I_{c}}{I_{c}+I_{a}}\times 100$$

where *X_c_* is the degree of crystallinity, *I_c_* is the intensity of the crystalline peak, and *I_a_* refers to the intensity of the amorphous peak. 

### 2.4. Mechanical Study

**Figure 4:**
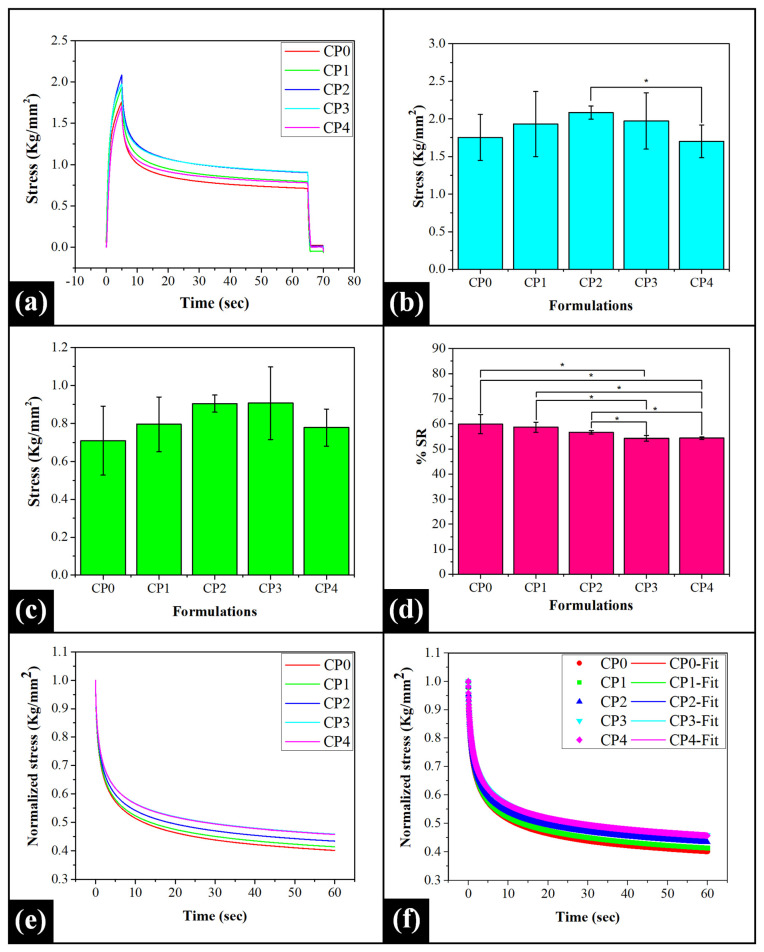
Mechanical study: (**a**) Stress relaxation profiles; (**b**) σ_max_ (mean ± standard deviation); (**c**) σ_min_ (mean ± standard deviation); (**d**) %SR (mean ± standard deviation); (**e**) Normalized stress relaxation profiles, and (**f**) Wiechert model fitting. The symbol * denotes the significant differences at *p* < 0.05 level.

The typical stress relaxation profiles of the prepared films demonstrated an increase in stress with time during the extension phase. At the end of the extension phase, the stress reached a maximum (σ_max_) value (Figure 4a; Table S2). The σ_max_ value represents the firmness of the films [71]. It can be observed that the firmness of CP0 and the remaining PVA/COS films was similar (*p* > 0.05) (Figure 4b). Among PVA/COS composite films, the σ_max_ value of CP1 was not significantly different from that of the other films (CP2, CP3, and CP4). The firmness of CP2 and CP3 was also similar (*p* > 0.05). However, the firmness of CP2 was higher than that of CP4 (*p* < 0.05). The firm structure of CP2 can be reasoned with the high level of intermolecular hydrogen bonding within its matrix, as was observed from the FTIR studies. Additionally, the differences in the firmness of CP4 were not statistically significant to CP1, CP2, and CP3 (*p* > 0.05). 

As per the stress relaxation profiles, the attainment of maximum stress (σ_max_) was followed by an exponential stress decay. After the relaxation process, the stress was reduced to a minimum value (σ_min_). The residual stress (σ_min_) obtained after the experiment represents the retained elastic component of the films [71]. The variations in the residual stress of the films have been represented in Figure 4c (Table S2). As compared to CP0, the residual elastic component of the PVA/COS films demonstrated no statistically significant differences (*p* > 0.05). The σ_min_ value of CP1 was similar to that of CP2, CP3, and CP4. Moreover, no statistically significant differences could be observed in the σ_min_ values of CP2, CP3, and CP4 (*p* > 0.05). However, the standard deviation of the σ_min_ value of CP2 was lower than CP3 and CP4. This suggested the formation of a homogenous matrix in CP2, which can explain the closely repeatable experimental values. The percentage relaxation of the stress (%SR) in the prepared films under strained conditions was calculated (Equation (6)) [71].

(6) $$\%\mathrm{SR}=\frac{\sigma_{\max}-{\;\sigma }_{\min}}{\sigma_{\max}}\times 100$$

where σ_max_ denotes the maximum stress attained during the extension stage (kg/mm^2^) and σ_min_ is the residual stress at the end of the relaxation process (kg/mm^2^).

The %SR parameter sheds light on the viscous and elastic nature of the films. Its value varies between 0–100%, where 0% SR value indicates an ideal elastic sample while a perfectly viscous or liquid sample exhibits 100% SR value. It can be observed that the %SR values of the prepared films varied within the range of 50–60%, which characteristically represented the viscoelastic nature of the film matrices (Figure 4d; Table S2). The %SR values of CP0 were similar to CP1 and CP2 (*p* > 0.05) while being significantly higher than CP3 and CP4 (*p* < 0.05). In the case of PVA/COS films, CP1 and CP2 exhibited similar %SR values (*p* > 0.05). However, as compared to CP1 and CP2, the %SR values decreased significantly in CP3 and CP4 (*p* < 0.05). This observation suggested that the elastic component of CP3 and CP4 was comparatively higher than the rest of the films. It should be noted that the %SR values of CP3 and CP4 were however similar (*p* > 0.05). 

The relaxation profile was then normalized (Figure 4e) and subsequently fitted to the Wiechert model of viscoelasticity (Equation 7). The model fitting relaxation profiles and parameters thereof have been represented in Figure 4f and Table S2, respectively. The P_0_ parameter represents the retained elastic energy within the films after the relaxation process [72]. CP0, CP1, and CP2 had identical P_0_ values (*p* > 0.05). However, the P_0_ value of CP0 was significantly lower than CP3 and CP4 (*p* < 0.05). Among PVA/COS films, the variations in the P_0_ value of CP1 and CP2 were similar (*p* > 0.05). However, as compared to both CP1 and CP2, the residual elastic component of CP3 and CP4 was significantly higher (*p* < 0.05). However, the P_0_ values of CP3 and CP4 were found to be similar (*p* > 0.05). The model parameter τ_1_ is regarded as the ’instantaneous relaxation time’ and indicates the rearrangements of the polymer molecules under stress conditions. As observed, the differences in the τ_1_ values of the films ranging from CP0 to CP3 were not statistically significant (*p* > 0.05). However, the τ_1_ value of CP0 was significantly lower than the τ_1_ value of CP4 (*p* < 0.05). This observation suggested that the incorporation of COS in higher amounts restricted the molecular rearrangements of the polymer molecules in CP4. It might have occurred due to the efficient transfer of the applied stress across the polymeric chains. A significantly faster dissipation of stress (lower τ_1_ value) was noticed in CP1 and CP2 as compared to CP4 (*p* < 0.05). The observed τ_1_ values for CP3 and CP4 were similar (*p* > 0.05). The delayed relaxation time (τ_2_) marks the breakage of the polymeric chains when exposed to stress conditions for longer durations. The variations in the τ_2_ values of CP0, CP1, and CP2 were not statistically significant (*p* > 0.05). The τ_2_ values of CP1 and CP2 were noticed to be similar (*p* > 0.05). However, as the COS concentration gradually increased in CP3 and CP4, the τ_2_ values of these blends increased significantly as compared to CP0 (*p* < 0.05). This suggested the potential of blend films CP3 and CP4 to endure stress conditions for longer durations without any structural breakage. The τ_2_ values of blend films CP1 and CP2 were lower than those of CP3 and CP4 (*p* < 0.05). Nevertheless, the increment in COS concentration had no significant effect on the τ_2_ values of CP3 and CP4 (*p* > 0.05).

(7) $$P\left(t\right)={\;P}_{0}+{\;P}_{1}\times {\;e\;}^{\left(-t/\tau_{1}\right)}+{\;P}_{2}\times {\;e\;}^{\left(-t/\tau_{2}\right)}$$

where P_0_, P_1_, and P_2_ are the spring constants, and τ_1_, τ_2_ are time constants of the dashpots (sec).

### 2.5. Differential Scanning Calorimetry (DSC) Analysis 

**Figure 5:**
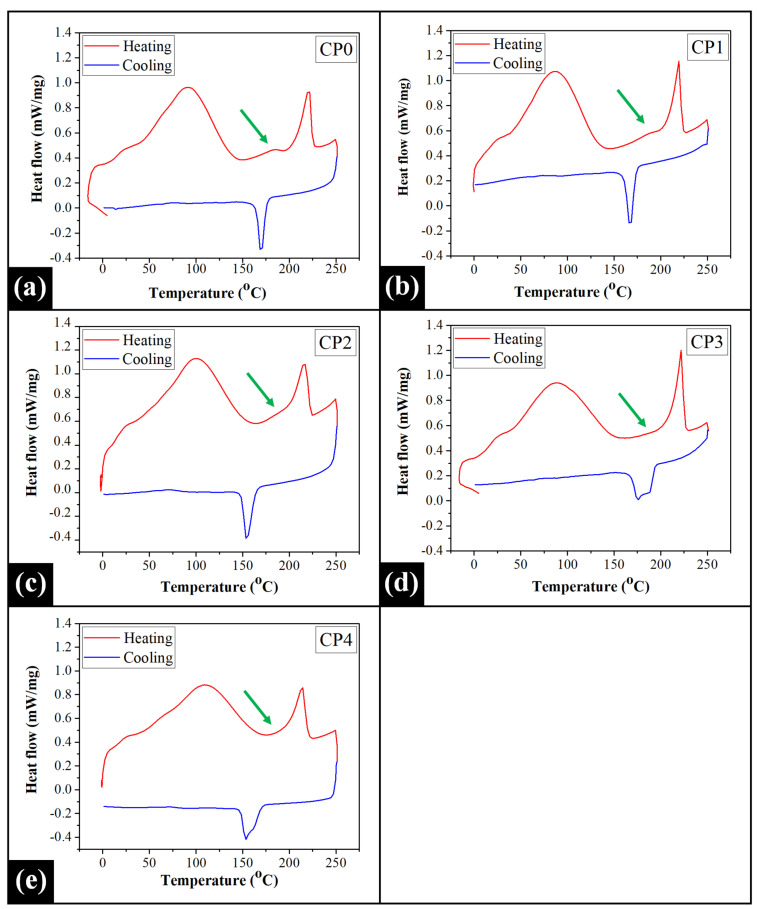
DSC thermograms of the prepared composite films: (**a**) CP0, (**b**) CP1, (**c**) CP2, (**d**) CP3, and (**e**) CP4.

The heating profiles of the prepared films demonstrated the presence of two or three distinct endothermic signals (Figure 5). The position of the peaks has been tabulated in Table S3. A broad endothermic peak was noticed at 90 °C in CP0. This peak is regarded as the "dehydration temperature" and has been attributed to the evaporation of the free water molecules (FWM) from the hydrophilic moieties of the polymers [73,74]. The inclusion of COS shifted the broad endothermic signal in CP1 to 87 °C. Interestingly, the removal of FWMs from the composite matrix of CP2 occurred at a comparatively higher temperature (99 °C). Afterward, an increment in the COS content caused a corresponding shifting of the endothermic signal to lower and higher temperatures in CP3 (88 °C) and CP4 (109 °C), respectively. The dehydration temperature (T_evap_) provides an insight regarding the strength of polymer-water interactions [73]. As observed, the occurrence of the highest T_evap_ in CP4 represented the strong hydrophilic interactions of FWMs with its composite matrix. The shifting in other samples might have arisen due to the alterations in the interaction between the PVA chains and COS, which ultimately caused physical and molecular changes. 

The control film also showed a hump-like endothermic peak in the heating profile at 184 °C, which indicated the removal of the bound water molecules from the PVA matrix (indicated by the green arrow). However, this signal was not distinctively observed in PVA/COS composite films. Closer observation showed that this signal fused with the adjacent sharp endothermic peak (recorded in the range of 214–221 °C). The fusion of the two peaks was becoming prominent with the corresponding increase in COS content. The sharp endothermic peak was observed at 222 °C in CP0, which has been documented as the melting temperature (T_m_) of PVA crystallites. The melting range of PVA recorded in this observation was in correlation with the previous reports [68,73]. As compared to CP0, the melting transition peak shifted to lower T_m_ values in CP1 (219 °C) and CP2 (217 °C). The shifting was also accompanied by the broadening of the sharp peak as the content of COS increased in these films. Some previously reported literature concerned with PVA and chitosan blend films has also reported the shifting of the PVA melting peak to lower temperatures with the addition of chitosan [75,76]. This shift in T_m_ values indicated good miscibility of the two polymers, which ultimately introduced alterations in the crystallization behavior of PVA. In other words, the admixing of COS slightly modified the crystal unit of PVA and hence, changed its melting profile. Interestingly, the T_m_ was observed at 222 °C in CP3, followed by an abrupt decline in CP4 (214 °C). The elevation in the T_m_ in CP3 can be ascribed to the presence of large-sized crystallites and the highly crystalline nature of the PVA matrix, as was observed from the diffraction studies. 

The thermogram of CP0 demonstrated a sharp exothermic peak at 169 °C in the cooling cycle, which can be attributed to the crystallization of PVA in the pristine PVA film. Surprisingly, the inclusion of COS in the polymer blend in different concentrations caused the shifting of this exothermic peak. It was observed that with an increment in COS content, the crystallization temperature (T_c_) reduced in CP1 (166 °C) and CP2 (153 °C). This shift in the T_c_ from higher to lower values, followed by the admixing of COS, suggested the reduction in the crystallization temperature of PVA. Interestingly, the crystallization of PVA occurred at a higher temperature (T_c_ = 176 °C) in CP3 while the peak acquired a wide shape as compared to the remaining films. Hence, it can be speculated that the addition of COS at this critical concentration (7.50 wt%) caused faster crystallization of PVA. Despite the broadening of the crystallization peak in CP4, the crystallization peak shifted to a lower temperature. The crystallization temperature of CP4 (T_c_ = 154 °C) was similar to that of CP2. 

### 2.6. Impedance Spectroscopy Analysis

**Figure 6:**
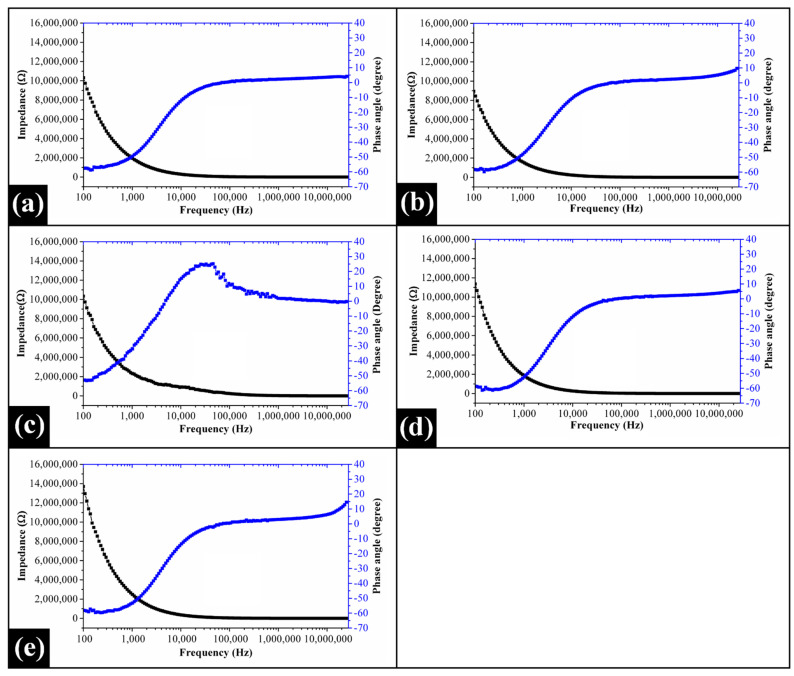
Electrical impedance profiles of the prepared films: (**a**) CP0, (**b**) CP1, (**c**) CP2, (**d**) CP3, and (**e**) CP4.

The impedance profiles of the prepared films featured the presence of two prominent regions, *viz.*, frequency-dependent region (<10 kHz), and frequency-independent plateau region (>10 kHz) (Figure 6). The electrical impedance of the films was relatively high at low frequencies, while the same exponentially degraded to a residual value at higher frequencies. This observation suggested the capacitive dominant nature of the films. At 100 Hz frequency, the impedance offered by the PVA matrix of CP0 to the flowing current was very high. The introduction of COS in the PVA matrix at an extremely low concentration (i.e., 2.50 wt%) decreased the electrical resistance of CP1. Interestingly, a monotonous increase in the impedance values was noticed in CP2, CP3, and CP4 with a corresponding increment in the COS content. The low values of the electrical conductivity of the films at low frequencies might be associated with the space charge polarization phenomenon at the electrode-film interface. This interfacial polarization occurs due to the deposition of the charge carriers (ionic species) near the electrode surfaces. This building up of charge carriers increases the dielectric constant of the materials, which thereby reduces the electrical conductivity. Contrastingly, at higher frequencies, the direction of the electrical field changes too quickly to provide enough time for these charge carriers to accumulate and align themselves in the direction of the electrical field. This causes a decline in the dielectric constant, which ultimately increases the conductivity [77,78,79]. Hence, the impedance spectra suggested that as the proportion of COS increased beyond 2.50 wt% (CP1), the conductivity of the PVA/COS films (CP2-CP4) reduced considerably. The variations in the magnitude of the phase angles (Φ) of the films as a function of current frequency were also recorded (Figure 6). Φ between the voltage and current has been reported to be –90° for ideal capacitors while the same is 0° for inductors [80]. In our study, at 100 Hz frequency, the Φ value for CP0 was observed to be –57.64°. In the case of CP1, the angle slightly shifted to a lower value of –58.20°. A sudden increment in the Φ value (–53.04°) was noticed for CP2. At this frequency, CP3 exhibited the lowest Φ value (–58.61°), which indicated that the capacitive behavior of this film was profound as compared to the remaining ones. The Φ value of CP4 (–58.11°) was in between the Φ values of CP1 and CP2. As the frequency of the current was in the higher range, the phase angles of the films increased gradually. This is suggestive of the coexistence of both the capacitive and resistive behavior of the films.

### 2.7. Antimicrobial Study

**Figure 7:**
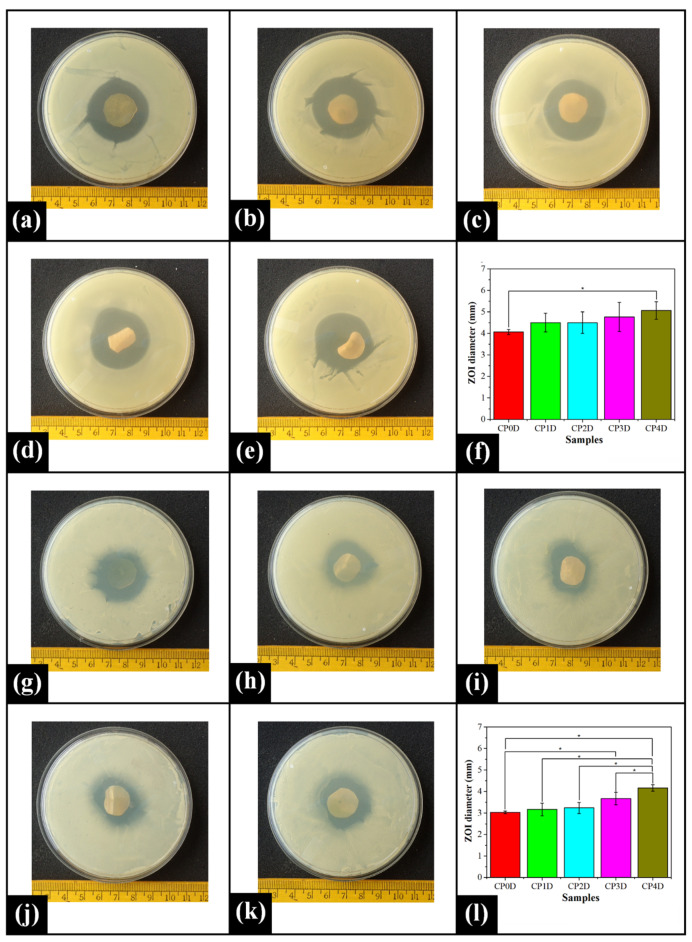
Antimicrobial activity of the CPH-loaded composite films against *E. coli*: (**a**) CP0D, (**b**) CP1D, (**c**) CP2D, (**d**) CP3D, (**e**) CP4D, (**f**) ZOI diameter (mean ± standard deviation); *B. cereus*; (**g**) CP0D, (**h**) CP1D, (**i**) CP2D, (**j**) CP3D, (**k**) CP4D, and (**l**) ZOI diameter (mean ± standard deviation). The symbol * denotes the significant differences at *p* < 0.05 level.

CPH is one of the most widely used fluoroquinolone antibiotics. It is a broad-spectrum antimicrobial drug to which Gram-positive and Gram-negative microorganisms are highly susceptible [81,82]. CPH inhibits prominent enzymes (such as DNA gyrase (topoisomerase II and topoisomerase IV) that are critical for bacterial DNA replication and thereby inhibit cell division [83,84]. The control films (without CPH) demonstrated no antimicrobial activity against the model organisms, i.e., *Escherichia coli* (*E. coli*) and *Bacillus cereus* (*B. cereus*) (Figure S3). However, CPH-loaded films showed clear ZOI against both, *E. coli* (Figure 7a–e) and *B. cereus* (Figure 7j–k). The ZOI diameter of CP0D, CP1D, CP2D, and CP3D against *E. coli* was observed to be similar (*p* > 0.05) (Figure 7f). However, the ZOI diameter in CP4D was significantly larger as compared to that of CP0D. Among the drug-loaded PVA/COS films, no significant variations in the ZOI diameter were observed (*p* > 0.05). In the case of *B. cereus*, the composite films, namely, CP0D, CP1D, and CP2D showed similar ZOI diameter (*p* > 0.05) (Figure 7l). As the COS content increased in CP3D and CP4D, the ZOI diameter in these samples increased significantly in comparison to CP0D (*p* < 0.05). Amongst PVA/COS films, the diameter of the inhibition zone acquired for CP1D, CP2D, and CP3D was similar (*p* > 0.05). CP4D exhibited the largest ZOI diameter against *B. cereus* among all drug-loaded films (*p* < 0.05). As per the above-mentioned results, it can be concluded that the drug-loaded films displayed excellent antimicrobial activity against both model organisms. This observation indicated that the prepared films can potentially be investigated as drug delivery matrices. 

### 2.8. Hemocompatibility Test

Blood compatibility is one of the major attributes demanded in polymeric matrices for biomedical applications [85]. It can be observed that the % hemolysis values of the tested films varied in the range of 0.034–0.256% (Table 2), which was much lower than 5%. As per the reported literature, the materials that demonstrate % hemolysis less than 5% are considered to be highly hemocompatible [86]. This indicated that all the tested films were non-hemolytic in nature, which reflected the blood compatible and biocompatible behavior of the same. The low % hemolysis values of the prepared films can be attributed to the highly hydrophilic nature of the constituent polymers, i.e., PVA and COS [87,88]. The hydrophilic nature of these polymers might have reduced polymer-red blood corpuscles (RBCs) interactions, which ultimately prevented the disruption of RBCs [87]. 

## 3. Conclusions

In the present study, we prepared novel PVA and COS-based blend films, which were crosslinked with glutaraldehyde. The prepared films were physicochemically and biologically characterized using several techniques. The introduction of COS as a co-ingredient changed the appearance of the PVA matrix from transparent and colorless (observed in CP0) to translucent and brown-colored in PVA/COS films. The FTIR spectroscopy revealed that the variation in COS content morphed the local environment of the PVA matrix in the blend films. The extent of hydrogen bonding was observed to be the highest in CP2 (5.0 wt% COS) as compared to the remaining films. XRD study demonstrated that the crystalline nature of the PVA/COS films (except CP1) was considerably higher than CP0. As compared to other films, the average crystallite size was the largest in CP3. Other parameters, such as relative crystallinity and degree of crystallinity, also indicated that the network structure of CP3 was highly ordered and crystalline. The thermal analysis confirmed the presence of different states of water (free and bound) in the prepared films. Additionally, the inclusion of COS enhanced the mechanical behavior of the PVA matrix as compared to that of CP0. PVA/COS films containing 7.50 wt% and 10.0 wt% (i.e., CP3 and CP4) of COS demonstrated low molecular rearrangements and polymer chain breakage under prolonged stress conditions. Impedance spectra revealed the capacitive dominant behavior of the films. Moreover, PVA/COS films (except CP1) possessed better dielectric properties than CP0. In contrast to the pristine films, CPH-loaded films demonstrated good antibacterial activity against the model organisms. The hemocompatibility test demonstrated the exceptional biocompatibility of the prepared films. Therefore, the new PVA/COS films can be proposed for application as biocompatible matrices for sustained drug delivery.

## 4. Materials and Methods

### 4.1. Materials

PVA (Average Molecular weight: 125,000; Degree of hydrolysis: 99%) was obtained from Loba Chemie Pvt. Ltd., Mumbai, India. COS gel (10% *w*/*w*) was purchased from Everest Biotech, Bangalore, India. GTA (25% pure) and hydrochloric acid (HCl; 35% pure) were obtained from Loba Chemie Pvt. Ltd., Mumbai, India. CPH was a kind gift obtained from Aristo Pharmaceutical, Bhopal, India. The microbial strains, namely, *E. coli* (MTCC443) and *B. cereus* (MTCC430), were procured from Microbial type culture collection and gene bank (MTCC), Chandigarh, India. Ethanol was purchased from Changshu Yangyuan Chemical, China. Brain heart infusion (BHI) agar and BHI nutrient broth were purchased from HiMedia Laboratories Pvt. Ltd., Mumbai, India. The reagents were used as received without any further processing. Fresh goat blood was procured from a local butcher shop. Double distilled water was used throughout the study.

### 4.2. Methods

#### 4.2.1. Preparation of Films

PVA solution (10 wt%) was prepared by dissolving 10 g of PVA in 90 g of water at 90 °C. A series of aqueous COS solutions were prepared by diluting the procured COS gel with water. The COS solutions contained 2.5%, 5.0%, 7.5%, and 10.0% w/w of COS in water. The PVA/COS films were developed by the solution casting method. In brief, 18 g of PVA solution and 2 g of COS solutions were mixed at 60 °C while maintaining continuous stirring conditions (200 rpm; 5 min); 20 g of water was then added to the prepared mixture, and the whole mixture was subsequently homogenized for 5 min, followed by degassing for 30 min. After degassing, 2 mL of 6, crosslinker (composition: 10 mL ethanol, 10 mL GTA, 0.1 mL HCl) was added to the above mixture and homogenized on a stirrer for 5 min. The mixture was kept at room temperature (25 ± 1 °C) for 30 min. Subsequently, 20 g of the obtained mixture was cast into the Petri plates (diameter: 9 cm) and dried at room temperature. After the films have been thoroughly dried, it was kept in airtight zip-lock packets. The films made with 2.5%, 5.0%, 7.5%, and 10.0% (*w*/*w*) COS solutions were named as CP1, CP2, CP3, and CP4, respectively. PVA film without COS was used as the control (CP0). For the synthesis of drug-loaded films, the polymer mixtures were prepared by following the aforementioned procedure. However, 170 mg of CPH was loaded and dissolved in the PVA/COS mixture prior to the addition of the crosslinker. CPH-loaded films were named CP0D, CP1D, CP2D, CP3D, and CP4D, respectively. The composition of the prepared films has been given in Table 3.

#### 4.2.2. FTIR Spectroscopy Analysis

FTIR spectra of the pristine PVA and PVA/COS blend films were recorded using an FTIR spectrometer (Alpha-E, Bruker, Billerica, MA, USA) attached with ZnSe crystal. The spectra of the samples were acquired in attenuated total reflectance (ATR) mode in the wavenumber range of 4000–400 cm^−1^. For all samples, the FTIR spectra were collected using 24 scans at 4 cm^−1^ spectral resolution [89]. 

#### 4.2.3. XRD Study

The diffraction profiles of the prepared films were obtained using an X-ray diffractometer (model: D8 Advance; Bruker, Billerica, MA, USA). The instrument used a cobalt target (Co-Kα; 1.79Å) for X-ray generation (operated at 40 kV and 40 mA). The films were cut into square pieces, having a length of 10 mm, which were then mounted over the sample holder. The diffractograms were recorded in the range of 5–50° 2θ while the scan rate was maintained at 5° 2θ/min.

#### 4.2.4. Mechanical Study 

The stress relaxation test of the films was performed using a static mechanical tester (Stable Micro Systems, TA-HD plus, Haslemere, UK) in extension mode. All the film samples were cut into rectangular pieces (dimension: 50 mm × 5 mm) and fixed in sample holders such that the window length was 40 mm. The experiment was done by stretching the films at an extension rate of 1 mm/sec up to a distance of 5 mm. The probe was maintained in the same position for 60 s and the films were allowed to relax during these strained conditions. The analysis was performed in triplicate for each film sample. 

#### 4.2.5. DSC Analysis

The differential scanning calorimeter (200 F3 DSC, Maia, Netzsch, Selb, Germany) was used for conducting the DSC analysis of the representative samples of each film. A small portion of the selected films (~2 mg) was cut and hermetically sealed in aluminum pans with pierced lids. An empty hermetically sealed aluminum pan with a pierced lid was used as the reference for the study. The analysis was done under an inert atmosphere of nitrogen gas. The heating curve was recorded by heating the films in the range of 0 °C and 250 °C. At 250 °C, there was a hold time of 5 min, and subsequently, the samples were cooled down to 0 °C. The thermal scanning rate was set at 10 °C/min, which was constant for the whole thermal program.

#### 4.2.6. Impedance Spectroscopy Analysis

The electrical impedance analysis of the prepared films was performed using an impedance analyzer (Digilent analog discovery 2, National Instruments, Austin, TX, USA). The films were cut into circular pieces of 10 mm diameter, which were then sandwiched between stainless steel electrodes. Subsequently, the impedance of the films was noted in the frequency range of 100 Hz and 25,000 kHz. 

#### 4.2.7. Antimicrobial Study

The antimicrobial effect of the control (without drug) and CPH-loaded PVA/COS films was assessed against two model organisms, namely, *B. cereus* (Gram-positive) and *E. coli* (Gram-negative), using the disc diffusion method. For the experiment, the prepared films were cut into discs (diameter: 15 mm); 200 µL aliquots of both bacterial strains (1 × 10^6^ CFU/ mL) were inoculated and spread on the surface of the BHI nutrient agar plates. Subsequently, the test film discs were placed onto the inoculated surface of the Petri plates. The plates were covered with parafilm to avoid dehydration and then incubated at 37 °C for 8 h. After the incubation, the zone of inhibition (ZOI) surrounding the film discs was measured. 

#### 4.2.8. Hemocompatibility Test

Hemocompatibility of the prepared films was investigated to assess the potential of inducing hemolysis by the films. For this purpose, the fresh goat blood was collected in the presence of sodium citrate as the anticoagulant. A small piece of each film (2×2 cm) was equilibrated with 10 mL normal saline (0.9% *w*/*v*) for 1 h at 37 °C. Thereafter, 0.5 mL of the equilibrated medium was then incubated with 0.5 mL of diluted blood (4 mL citrated blood: 5 mL normal saline), and the final volume was adjusted to 10 mL with normal saline. The positive control was prepared by mixing 0.5 mL 0.1 N HCl (used in place of the test sample) with 0.5 mL diluted blood, which was then diluted to 10 mL using normal saline. On the other hand, 0.5 mL of diluted blood was added with normal saline (9.5 mL) to prepare a negative control. The test samples along with positive and negative controls were then incubated at 37 °C for 1 h. Thereafter, these samples were centrifuged at 3000 rpm for 10 min. The optical density (OD) of the supernatant was determined at 545 nm using a spectrophotometer (Shimadzu-1700, Tokyo, Japan). The test was conducted in triplicates for each sample. The hemolysis percentage was calculated using Equation (8) [85].

(8) $$\%\;\mathrm{Hemolysis}=\frac{{\mathrm{OD}}_{\mathrm{test}}-{\mathrm{OD}}_{\mathrm{negative}}}{{\mathrm{OD}}_{\mathrm{positive}}-{\mathrm{OD}}_{\mathrm{negative}}}\times 100$$

where OD_test_ is the absorbance of the tested film sample, OD_negative_ refers to the absorbance of the negative control, and OD_positive_ is the absorbance of the positive control. 

#### 4.2.9. Statistical Analysis 

In the case of analyses that were performed in triplicates, the data has been presented as mean ± standard deviation. The means were statistically analyzed by using student *t*-test at *p* < 0.05 significance level.

## Figures and Tables

**Table 1:** XRD peak parameters after deconvolution.

Samples	Peaks	Peak Position (x_c_) (° 2θ)	FWHM (° 2θ)	d-Spacing (Å)	Crystallite Size (D) (nm)	Lattice Strain
**CP0**	Peak A	22.699	7.104	4.545	1.380	0.154
	Peak B	22.699	1.877	4.545	5.240	0.041
	**Average**		**4.491**	**4.545**	**3.310**	**0.098**
**CP1**	Peak A	22.677	7.007	4.550	1.400	0.153
	Peak B	22.677	1.896	4.550	5.190	0.041
	**Average**		**4.452**	**4.550**	**3.295**	**0.097**
**CP2**	Peak A	22.871	7.305	4.512	1.350	0.158
	Peak B	22.871	1.906	4.512	5.160	0.041
	**Average**		**4.606**	**4.512**	**3.255**	**0.099**
**CP3**	Peak A	22.864	6.843	4.513	1.440	0.148
	Peak B	22.864	1.827	4.513	5.380	0.039
	**Average**		**4.335**	**4.513**	**3.410**	**0.094**
**CP4**	Peak A	22.789	7.104	4.528	1.380	0.154
	Peak B	22.789	1.904	4.528	5.160	0.041
	**Average**		**4.504**	**4.528**	**3.270**	**0.098**

**Table 2:** % Hemolysis values of the prepared films.

Formulations	% Hemolysis
**CP0**	0.256 ± 0.171
**CP1**	0.034 ± 0.085
**CP2**	0.057 ± 0.053
**CP3**	0.036 ± 0.073
**CP4**	0.115 ± 0.136

**Table 3:** Composition of the films.

Film Samples	PVA Solution (g; 10% *w*/*w*)	COS Solution (g)					Water (g)	Crosslinker Reagent (mL)	CPH (g)	wt% of COS in Films
		0% (water)	2.5%	5.0%	7.5%	10.0%				
CP0	18.00	2.00	--	--	--	--	20.00	2.00	0.00	0.00
CP1	18.00	--	2.00		--	--	20.00	2.00	0.00	2.70
CP2	18.00	--	--	2.00	--	--	20.00	2.00	0.00	5.40
CP3	18.00	--	--	--	2.00	--	20.00	2.00	0.00	8.11
CP4	18.00	--	--	--	--	2.00	20.00	2.00	0.00	10.81
CP0D	18.00	2.00	--	--	--	--	20.00	2.00	0.17	0.00
CP1D	18.00	--	2.00		--	--	20.00	2.00	0.17	2.70
CP2D	18.00	--	--	2.00	--	--	20.00	2.00	0.17	5.40
CP3D	18.00	--	--	--	2.00	--	20.00	2.00	0.17	8.11
CP4D	18.00	--	--	--	--	2.00	20.00	2.00	0.17	10.81

“--” corresponds to the absence of the constituent.

## Data Availability

The data presented in this study are available on request from the corresponding author.

## Supplementary Materials

The following are available online at https://www.mdpi.com/article/10.3390/gels7020055/s1. Figure S1: Area under the O-H stretching peak of the prepared films: (a) CP0, (b) CP1, (c) CP2, (d) CP3, and (e) CP4, Figure S2: Deconvoluted diffractograms of the prepared films: (a) CP0, (b) CP1, (c) CP2, (d) CP3, and (e) CP4, Table S1: Parameters calculated from the deconvoluted peaks, Table S2: Stress relaxation parameters, Table S3: DSC peak parameters, Figure S3: Antimicrobial activity of the pristine films (without drug) against *E. coli*: (a) CP0, (b) CP1, (c) CP2, (d) CP3, and (e) CP4; *B. cereus*: (f) CP0, (g) CP1, (h) CP2, (i) CP3, and (j) CP4.
