# Supplementary material for: Fabrication and Characterization of Poly (vinyl alcohol) and Chitosan Oligosaccharide-Based Blend Films
Source: Gels. 2021 May 6;7(2):55. doi: 10.3390/gels7020055 (PMC8162339; doi:10.3390/gels7020055)
Supplement: Supplementary file 1 [file gels-07-00055-s001.zip › gels-1068609-supplementary.pdf]

## *Supplementary file*

### 1. FTIR spectroscopy analysis

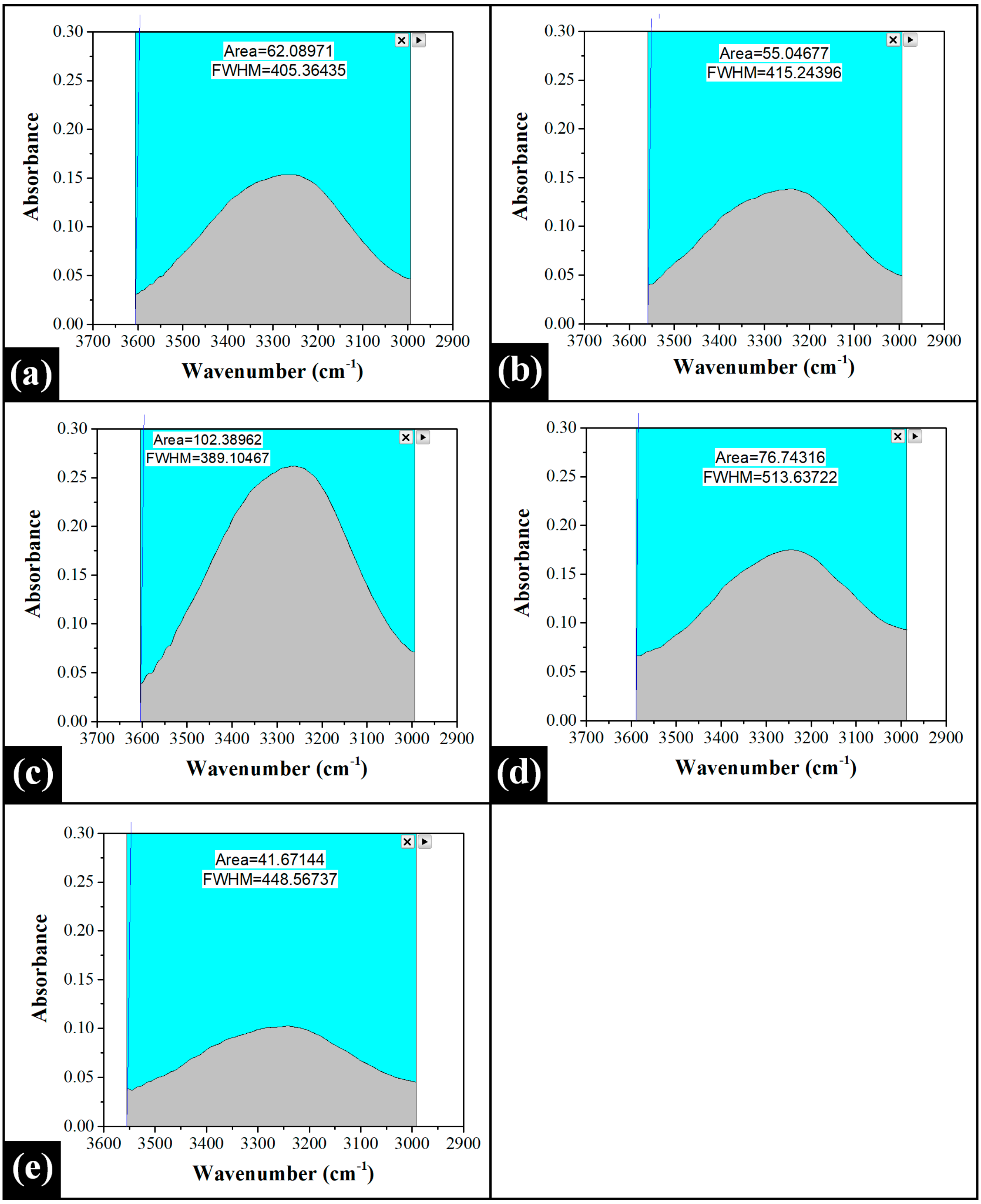


**Figure S1. Area under the O-H stretching peak of the prepared films: (a) CP0, (b) CP1, (c) CP2, (d) CP3, and (e) CP4.**

## 2. XRD analysis

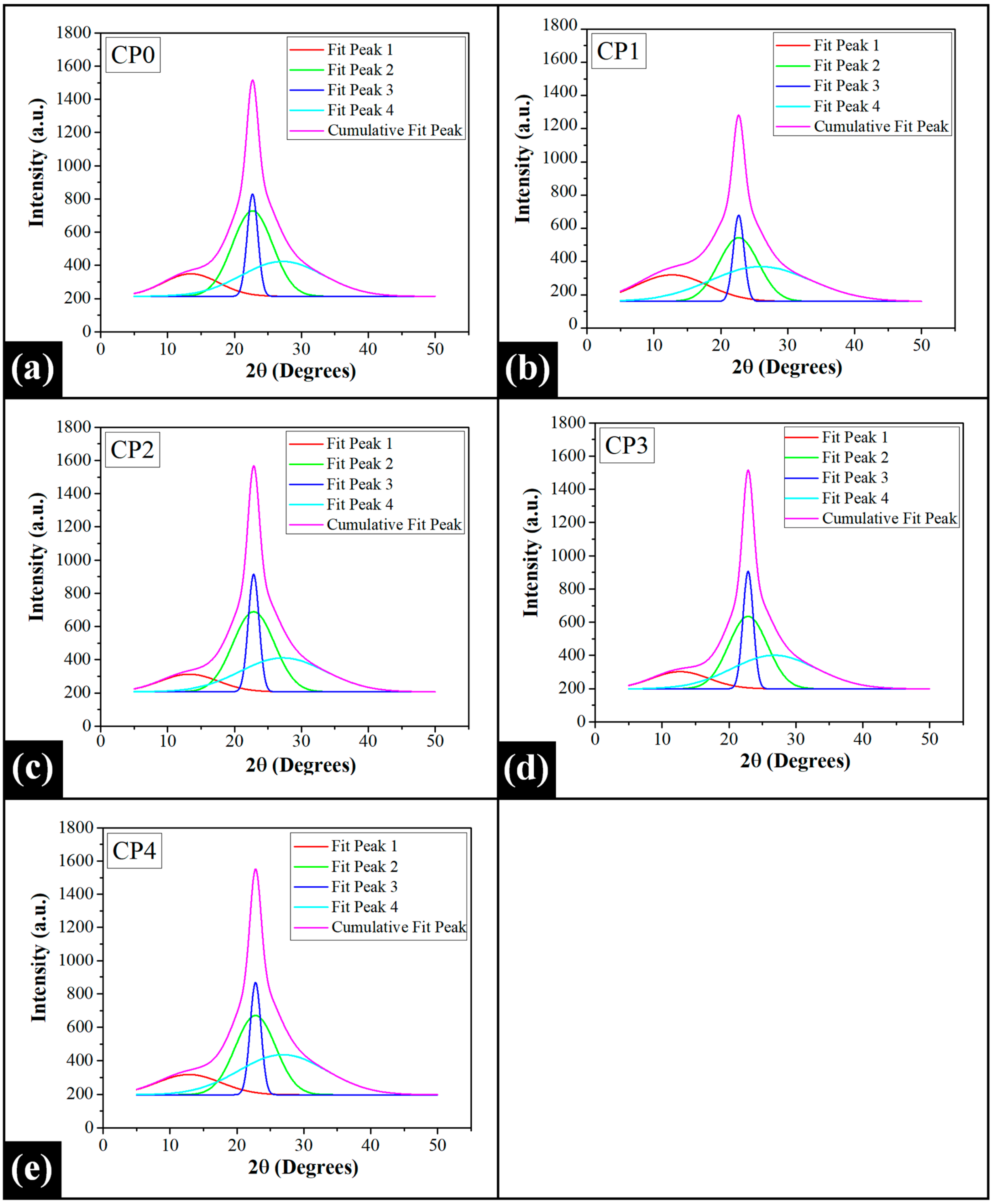


**Figure S2. Deconvoluted diffractograms of the prepared films: (a) CP0, (b) CP1, (c) CP2, (d) CP3, and (e) CP4.**

**Table S1. Parameters calculated from the deconvoluted peaks.**

| Samples | % Δcrystallinity | Peak height ratio | Degree of crystallinity ($X_c$) |
|---|---|---|---|
| **CP0** | -- | 1.196 | 53.232 |
| **CP1** | -14.511 | 1.352 | 55.525 |
| **CP2** | 4.381 | 1.467 | 57.021 |
| **CP3** | 2.603 | 1.619 | 58.773 |
| **CP4** | 3.911 | 1.417 | 56.416 |

**3. Stress relaxation analysis**

**Table S2. Stress relaxation parameters.**

| **Analysis Model** | **Parameters** | **Film samples** | | | | |
|---|---|---|---|---|---|---|
| | | **CP0** | **CP1** | **CP2** | **CP3** | **CP4** |
| **--** | $\sigma_{max}$ (Kg/mm$^2$) | 1.753 ± 0.307 | 1.932 ± 0.432 | 2.083 ± 0.088 | 1.972 ± 0.374 | 1.702 ± 0.217 |
| | $\sigma_{min}$ (Kg/mm$^2$) | 0.709 ± 0.181 | 0.794 ± 0.144 | 0.903 ± 0.045 | 0.906 ± 0.190 | 0.777 ± 0.097 |
| | %SR | 59.852 ± 3.837 | 58.613 ± 2.037 | 56.609 ± 0.712 | 54.179 ± 1.107 | 54.298 ± 0.442 |
| **Wiechert model** | $P_0$ | 0.401 ± 0.037 | 0.413 ± 0.020 | 0.433 ± 0.007 | 0.456 ± 0.010 | 0.455 ± 0.004 |
| | $P_1$ | 0.304 ± 0.014 | 0.302 ± 0.011 | 0.292 ± 0.004 | 0.275 ± 0.005 | 0.277 ± 0.004 |
| | $\tau_1$ (sec) | 1.254 ± 0.132 | 1.336 ± 0.036 | 1.377 ± 0.014 | 1.377 ± 0.082 | 1.430 ± 0.012 |
| | $P_2$ | 0.220 ± 0.022 | 0.208 ± 0.008 | 0.204 ± 0.002 | 0.199 ± 0.001 | 0.200 ± 0.001 |
| | $\tau_2$ (sec) | 15.888 ± 0.878 | 16.579 ± 0.260 | 16.849 ± 0.093 | 17.622 ± 0.364 | 17.549 ± 0.174 |
| | $R^2$ | 0.998 | 0.998 | 0.998 | 0.998 | 0.998 |

## 4. DSC analysis

**Table S3. DSC peak parameters**

| **Samples** | **$T_{evap}$ (ºC)** | **$T_m$ (ºC)** | **$T_c$ (ºC)** |
|---|---|---|---|
| **CP0** | 90 | 221 | 169 |
| **CP1** | 87 | 219 | 166 |
| **CP2** | 99 | 217 | 153 |
| **CP3** | 88 | 222 | 176 |
| **CP4** | 109 | 214 | 154 |

## 5. Antimicrobial analysis

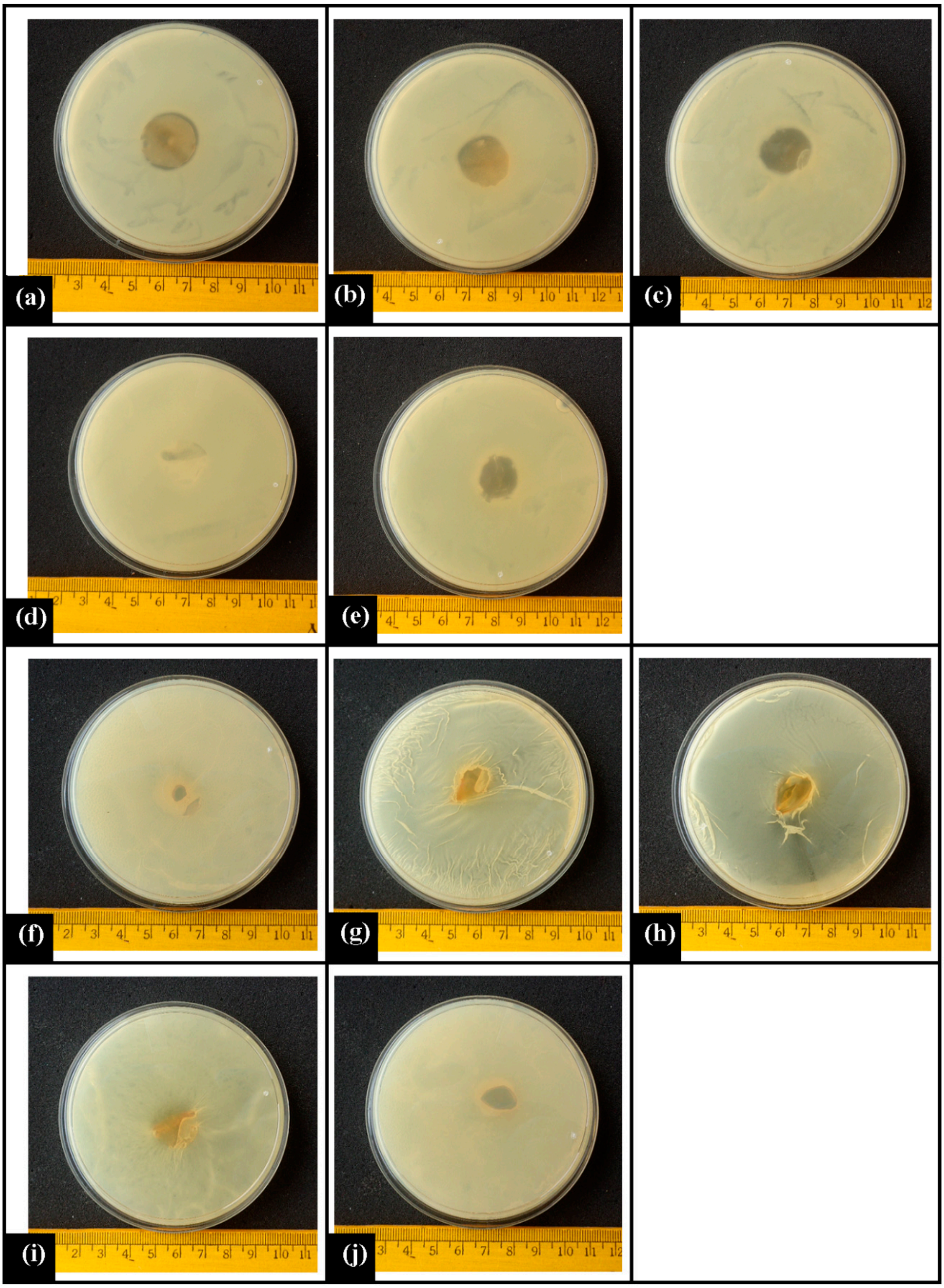

**Figure S3. Antimicrobial activity of the pristine films (without drug) against *E. coli*: (a) CP0, (b) CP1, (c) CP2, (d) CP3, (e) CP4; *B. cereus*: (f) CP0, (g) CP1, (h) CP2, (i) CP3, (j) CP4.**
